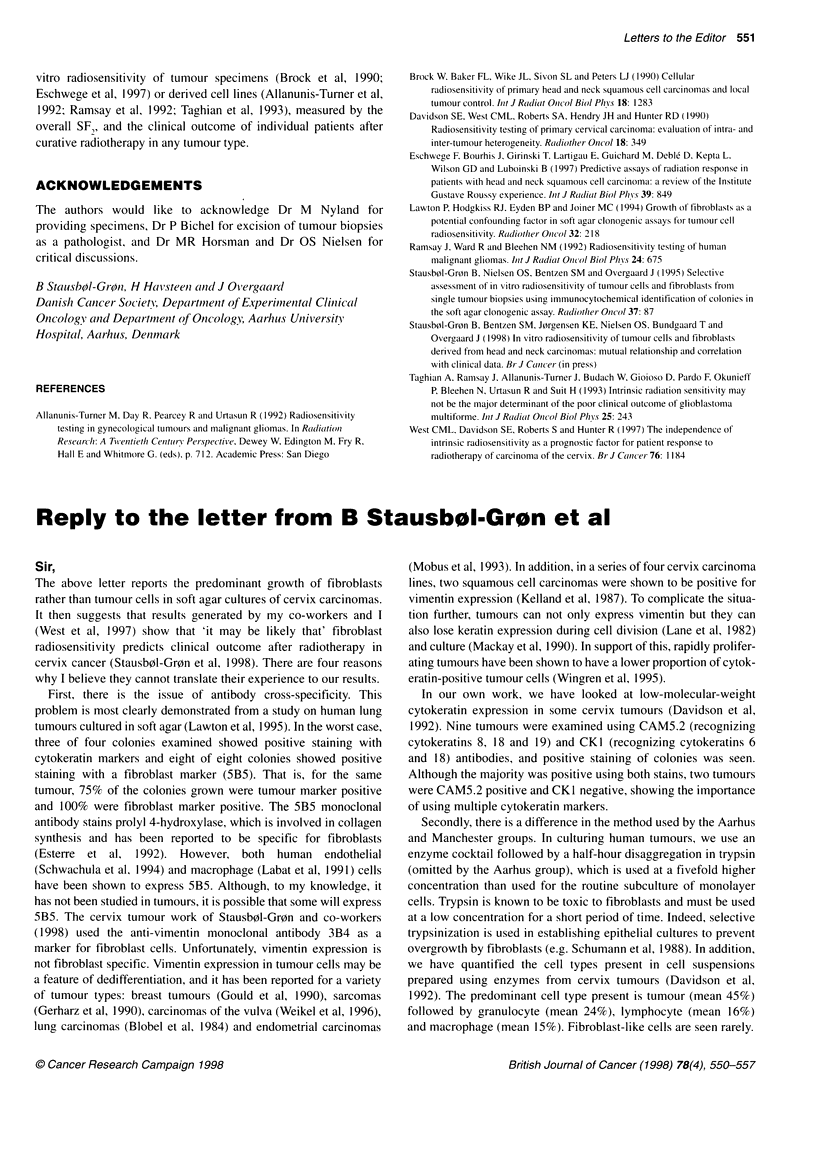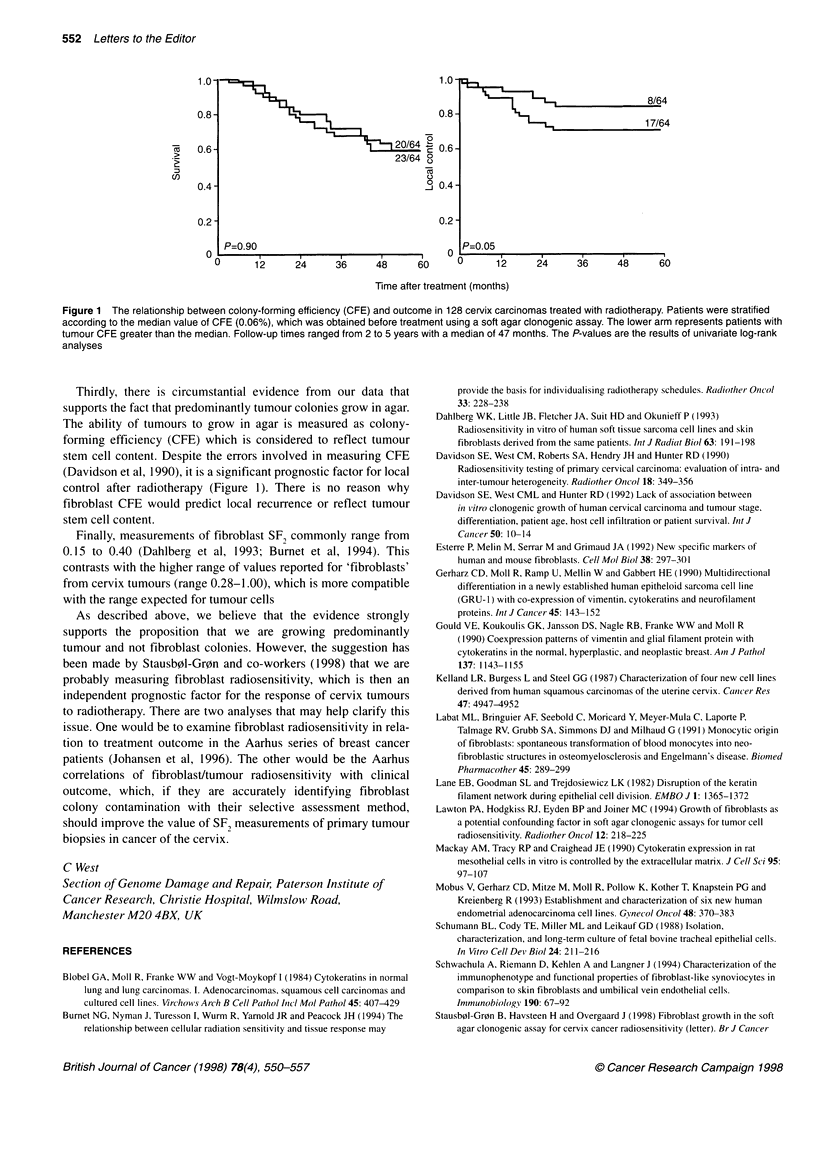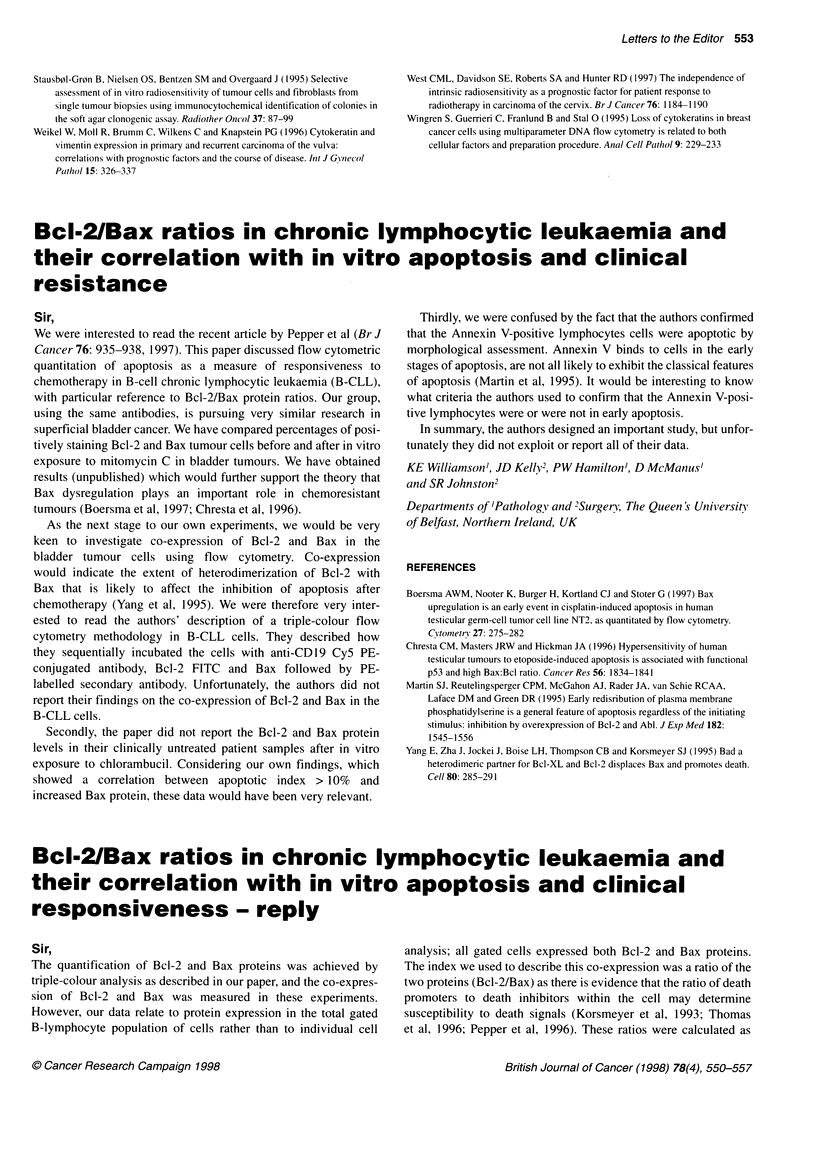# Reply to the letter from B Stausbøl-Grøn et al

**Published:** 1998-08

**Authors:** C West


### Reply to the letter from B Stausbøl-Grøn et al

**Sir,**

The above letter reports the predominant growth of fibroblasts rather than tumour cells in soft agar cultures of cervix carcinomas. It then suggests that results generated by my co-workers and I (West et al, 1997) show that 'it may be likely that' fibroblast radiosensitivity predicts clinical outcome after radiotherapy in cervix cancer (Stausbøl-Grøn et al, 1998). There are four reasons why I believe they cannot translate their experience to our results.

First, there is the issue of antibody cross-specificity. This problem is most clearly demonstrated from a study on human lung tumours cultured in soft agar (Lawton et al, 1995). In the worst case, three of four colonies examined showed positive staining with cytokeratin markers and eight of eight colonies showed positive staining with a fibroblast marker (5B5). That is, for the same tumour, 75% of the colonies grown were tumour marker positive and 100% were fibroblast marker positive. The 5B5 monoclonal antibody stains prolyl 4-hydroxylase, which is involved in collagen synthesis and has been reported to be specific for fibroblasts (Esterre et al, 1992). However, both human endothelial (Schwachula et al, 1994) and macrophage (Labat et al, 1991) cells have been shown to express 5B5. Although, to my knowledge, it has not been studied in tumours, it is possible that some will express 5B5. The cervix tumour work of Stausbøl-Grøn and co-workers (1998) used the anti-vimentin monoclonal antibody 3B4 as a marker for fibroblast cells. Unfortunately, vimentin expression is not fibroblast specific. Vimentin expression in tumour cells may be a feature of dedifferentiation, and it has been reported for a variety of tumour types: breast tumours (Gould et al, 1990), sarcomas (Gerharz et al, 1990), carcinomas of the vulva (Weikel et al, 1996), lung carcinomas (Blobel et al, 1984) and endometrial carcinomas (Mobus et al, 1993). In addition, in a series of four cervix carcinoma lines, two squamous cell carcinomas were shown to be positive for vimentin expression (Kelland et al, 1987). To complicate the situation further, tumours can not only express vimentin but they can also lose keratin expression during cell division (Lane et al, 1982) and culture (Mackay et al, 1990). In support of this, rapidly proliferating tumours have been shown to have a lower proportion of cytokeratin-positive tumour cells (Wingren et al, 1995).

In our own work, we have looked at low-molecular-weight cytokeratin expression in some cervix tumours (Davidson et al, 1992). Nine tumours were examined using CAM5.2 (recognizing cytokeratins 8, 18 and 19) and CK1 (recognizing cytokeratins 6 and 18) antibodies, and positive staining of colonies was seen. Although the majority was positive using both stains, two tumours were CAM5.2 positive and CK1 negative, showing the importance of using multiple cytokeratin markers.

Secondly, there is a difference in the method used by the Aarhus and Manchester groups. In culturing human tumours, we use an enzyme cocktail followed by a half-hour disaggregation in trypsin (omitted by the Aarhus group), which is used at a fivefold higher concentration than used for the routine subculture of monolayer cells. Trypsin is known to be toxic to fibroblasts and must be used at a low concentration for a short period of time. Indeed, selective trypsinization is used in establishing epithelial cultures to prevent overgrowth by fibroblasts (e.g. Schumann et al, 1988). In addition, we have quantified the cell types present in cell suspensions prepared using enzymes from cervix tumours (Davidson et al, 1992). The predominant cell type present is tumour (mean 45%) followed by granulocyte (mean 24%), lymphocyte (mean 16%) and macrophage (mean 15%). Fibroblast-like cells are seen rarely.

© Cancer Research Campaign 1998

**Figure 1** The relationship between colony-forming efficiency (CFE) and outcome in 128 cervix carcinomas treated with radiotherapy. Patients were stratified according to the median value of CFE (0.06%), which was obtained before treatment using a soft agar clonogenic assay. The lower arm represents patients with tumour CFE greater than the median. Follow-up times ranged from 2 to 5 years with a median of 47 months. The P-values are the results of univariate log-rank analyses

Thirdly, there is circumstantial evidence from our data that supports the fact that predominantly tumour colonies grow in agar. The ability of tumours to grow in agar is measured as colony-forming efficiency (CFE) which is considered to reflect tumour stem cell content. Despite the errors involved in measuring CFE (Davidson et al, 1990), it is a significant prognostic factor for local control after radiotherapy (Figure 1). There is no reason why fibroblast CFE would predict local recurrence or reflect tumour stem cell content.

Finally, measurements of fibroblast $SF_2$ commonly range from 0.15 to 0.40 (Dahlberg et al, 1993; Burnet et al, 1994). This contrasts with the higher range of values reported for 'fibroblasts' from cervix tumours (range 0.28–1.00), which is more compatible with the range expected for tumour cells

As described above, we believe that the evidence strongly supports the proposition that we are growing predominantly tumour and not fibroblast colonies. However, the suggestion has been made by Stausbøl-Grøn and co-workers (1998) that we are probably measuring fibroblast radiosensitivity, which is then an independent prognostic factor for the response of cervix tumours to radiotherapy. There are two analyses that may help clarify this issue. One would be to examine fibroblast radiosensitivity in relation to treatment outcome in the Aarhus series of breast cancer patients (Johansen et al, 1996). The other would be the Aarhus correlations of fibroblast/tumour radiosensitivity with clinical outcome, which, if they are accurately identifying fibroblast colony contamination with their selective assessment method, should improve the value of $SF_2$ measurements of primary tumour biopsies in cancer of the cervix.

*C West*
*Section of Genome Damage and Repair, Paterson Institute of Cancer Research, Christie Hospital, Wilmslow Road, Manchester M20 4BX, UK*

### REFERENCES

Blobel GA, Moll R, Franke WW and Vogt-Moykopf I (1984) Cytokeratins in normal lung and lung carcinomas. I. Adenocarcinomas, squamous cell carcinomas and cultured cell lines. *Virchows Arch B Cell Pathol Incl Mol Pathol* **45**: 407–429

Burnet NG, Nyman J, Turesson I, Wurm R, Yarnold JR and Peacock JH (1994) The relationship between cellular radiation sensitivity and tissue response may provide the basis for individualising radiotherapy schedules. *Radiother Oncol* **33**: 228–238

Dahlberg WK, Little JB, Fletcher JA, Suit HD and Okunieff P (1993) Radiosensitivity in vitro of human soft tissue sarcoma cell lines and skin fibroblasts derived from the same patients. *Int J Radiat Biol* **63**: 191–198

Davidson SE, West CM, Roberts SA, Hendry JH and Hunter RD (1990) Radiosensitivity testing of primary cervical carcinoma: evaluation of intra- and inter-tumour heterogeneity. *Radiother Oncol* **18**: 349–356

Davidson SE, West CML and Hunter RD (1992) Lack of association between *in vitro* clonogenic growth of human cervical carcinoma and tumour stage, differentiation, patient age, host cell infiltration or patient survival. *Int J Cancer* **50**: 10–14

Esterre P, Melin M, Serrar M and Grimaud JA (1992) New specific markers of human and mouse fibroblasts. *Cell Mol Biol* **38**: 297–301

Gerharz CD, Moll R, Ramp U, Mellin W and Gabbert HE (1990) Multidirectional differentiation in a newly established human epitheloid sarcoma cell line (GRU-1) with co-expression of vimentin, cytokeratins and neurofilament proteins. *Int J Cancer* **45**: 143–152

Gould VE, Koukoulis GK, Jansson DS, Nagle RB, Franke WW and Moll R (1990) Coexpression patterns of vimentin and glial filament protein with cytokeratins in the normal, hyperplastic, and neoplastic breast. *Am J Pathol* **137**: 1143–1155

Kelland LR, Burgess L and Steel GG (1987) Characterization of four new cell lines derived from human squamous carcinomas of the uterine cervix. *Cancer Res* **47**: 4947–4952

Labat ML, Bringuier AF, Seebold C, Moricard Y, Meyer-Mula C, Laporte P, Talmage RV, Grubb SA, Simmons DJ and Milhaud G (1991) Monocytic origin of fibroblasts: spontaneous transformation of blood monocytes into neo-fibroblastic structures in osteomyelosclerosis and Engelmann's disease. *Biomed Pharmacother* **45**: 289–299

Lane EB, Goodman SL and Trejdosiewicz LK (1982) Disruption of the keratin filament network during epithelial cell division. *EMBO J* **1**: 1365–1372

Lawton PA, Hodgkiss RJ, Eyden BP and Joiner MC (1994) Growth of fibroblasts as a potential confounding factor in soft agar clonogenic assays for tumor cell radiosensitivity. *Radiother Oncol* **12**: 218–225

Mackay AM, Tracy RP and Craighead JE (1990) Cytokeratin expression in rat mesothelial cells in vitro is controlled by the extracellular matrix. *J Cell Sci* **95**: 97–107

Mobus V, Gerharz CD, Mitze M, Moll R, Pollow K, Kother T, Knapstein PG and Kreienberg R (1993) Establishment and characterization of six new human endometrial adenocarcinoma cell lines. *Gynecol Oncol* **48**: 370–383

Schumann BL, Cody TE, Miller ML and Leikauf GD (1988) Isolation, characterization, and long-term culture of fetal bovine tracheal epithelial cells. *In Vitro Cell Dev Biol* **24**: 211–216

Schwachula A, Riemann D, Kehlen A and Langner J (1994) Characterization of the immunophenotype and functional properties of fibroblast-like synoviocytes in comparison to skin fibroblasts and umbilical vein endothelial cells. *Immunobiology* **190**: 67–92

Stausbøl-Grøn B, Havsteen H and Overgaard J (1998) Fibroblast growth in the soft agar clonogenic assay for cervix cancer radiosensitivity (letter). *Br J Cancer*

© Cancer Research Campaign 1998

Stausbøl-Grøn B, Nielsen OS, Bentzen SM and Overgaard J (1995) Selective assessment of in vitro radiosensitivity of tumour cells and fibroblasts from single tumour biopsies using immunocytochemical identification of colonies in the soft agar clonogenic assay. *Radiother Oncol* **37**: 87–99

Weikel W, Moll R, Brumm C, Wilkens C and Knapstein PG (1996) Cytokeratin and vimentin expression in primary and recurrent carcinoma of the vulva: correlations with prognostic factors and the course of disease. *Int J Gynecol Pathol* **15**: 326–337

West CML, Davidson SE, Roberts SA and Hunter RD (1997) The independence of intrinsic radiosensitivity as a prognostic factor for patient response to radiotherapy in carcinoma of the cervix. *Br J Cancer* **76**: 1184–1190

Wingren S, Guerrieri C, Franlund B and Stal O (1995) Loss of cytokeratins in breast cancer cells using multiparameter DNA flow cytometry is related to both cellular factors and preparation procedure. *Anal Cell Pathol* **9**: 229–233